# Metabolic Reprogramming by Andrographolide: Enhanced Pentose Phosphate Pathway and Antioxidant Capacity in Cortical Astrocytes

**DOI:** 10.3390/ph19010133

**Published:** 2026-01-12

**Authors:** Pedro Cisternas, Paulina Ormazabal, Camila Gherardelli, Marianela Bastías-Pérez, Jose Brito-Valenzuela, Nibaldo C. Inestrosa

**Affiliations:** 1 Núcleo de Investigación en Nutrición y Ciencias Alimentarias (NINCAL), Facultad de Salud y Ciencias Sociales, Universidad de Las Américas, Santiago 7500658, Chile; 2 Facultad de Ciencias para el Cuidado de la Salud, Universidad San Sebastián, Lota 2465, Providencia, Santiago 7510157, Chile; 3 Facultad de Ciencias Biológicas, Pontificia Universidad Católica de Chile, Av. Bernardo O’Higgins 340, Santiago 8331150, Chile; 4 Centro de Investigación Biomédica en Red (CIBER) de Fisiopatología de la Obesidad y Nutrición (CIBEROBN), Instituto de Salud Carlos III (ISCIII), E-28029 Madrid, Spain; 5 Departamento de Obstetricia y Puericultura, Facultad de Medicina, Universidad de Concepción, Concepción 4030000, Chile; 6 Centro de Excelencia en Biomedicina de Magallanes (CEBIMA), Escuela de Medicina, Universidad de Magallanes, Punta Arenas 6200000, Chile

**Keywords:** astrocytes, andrographolide, glucose metabolism, neuroprotection, Alzheimer disease

## Abstract

**Background/Objectives:** Astrocytes are key regulators of brain energy homeostasis, integrating glucose metabolism with antioxidant support for neuronal function. Dysregulation of these processes contributes to neurodegenerative diseases, including Alzheimer’s disease. Andrographolide, a bioactive diterpenoid from *Andrographis paniculata*, has been reported to exert neuroprotective effects through the modulation of Wnt/β–catenin signaling and neuronal metabolism; however, its actions on astrocytic metabolic pathways remain insufficiently characterized. **Methods:** Here, we investigated the effects of andrographolide on metabolic and redox parameters in primary mouse cortical astrocytes. **Results:** Andrographolide increased glucose uptake and antioxidant capacity without affecting AMPK activation or the activity of core glycolytic enzymes. Instead, it selectively enhanced glucose-6-phosphate dehydrogenase activity, promoting glucose flux through the pentose phosphate pathway in a partially Wnt-dependent manner. This metabolic reprogramming was associated with increased NADPH availability and glutathione levels, together with a reduced ATP/ADP ratio, consistent with a shift toward redox maintenance rather than maximal energy production. **Conclusions:** Collectively, these findings highlight astrocytic metabolic plasticity as a relevant and underexplored target of andrographolide and support the concept that natural compounds can enhance brain resilience by modulating glial redox metabolism.

## 1. Introduction

Brain glucose metabolism is fundamental to neuronal viability, synaptic activity, and redox balance [1,2]. While neurons exhibit high energetic requirements, astrocytes orchestrate cerebral glucose handling by regulating its uptake, storage, and metabolic redistribution, thereby sustaining neuronal function through lactate supply and glutathione (GSH) production [3,4,5]. Following cellular uptake, glucose is primarily directed toward glycolysis to support ATP generation or diverted to the pentose phosphate pathway (PPP), which provides reducing equivalents and biosynthetic intermediates [6,7].

In astrocytes, the PPP plays a critical role in redox regulation. NADPH generated by glucose-6-phosphate dehydrogenase (G6PDH) fuels glutathione recycling and supports antioxidant defenses, including the detoxification of reactive oxygen species [8,9]. Disruption of astrocytic glucose metabolism and redox control has been linked to oxidative stress and metabolic vulnerability in neurodegenerative disorders such as Alzheimer’s disease (AD), where impaired glial support exacerbates neuronal dysfunction [10,11,12].

Andrographolide (Andro), a labdane diterpenoid isolated from *Andrographis paniculata*, has gained attention for its anti-inflammatory, antioxidant, and neuroprotective properties [13,14]. In neurons has been described that Andro activates the canonical Wnt/β–catenin pathway promoting enhances glucose uptake and glycolytic activity through AMPK-dependent and Wnt-mediated mechanisms [15]. Despite the progress made in understanding Andro actions in the brain, how this compound shapes astrocyte-specific energy metabolism has remained insufficiently explored [13]. In this study, we focused on the early metabolic response of primary cortical astrocytes to Andro and found a pattern that differs from what has been described in neurons.

Our data show that Andro increases glucose uptake and promotes a selective activation of G6PDH and a shift toward PPP activity, reflected in higher NADPH/NADP^+^ ratios, increased GSH levels, and elevated GPx activity. Taken together, our results refine current knowledge by showing that Andro does not merely replicate previously described mechanisms, but extends them to the astrocyte context, revealing a cell-type-specific metabolic outcome: instead of enhancing glycolysis, Andro channels glucose toward the PPP to boost NADPH-dependent redox capacity and dampen inflammatory signaling. These findings broaden the understanding of Andro’s neuroprotective actions and highlight the potential relevance of glial metabolic remodeling in neurodegenerative conditions characterized by oxidative stress and impaired energy homeostasis, such as AD.

## 2. Results

### 2.1. Andrographolide Enhances Glucose Uptake in Cultured Astrocytes

**Figure 1:**
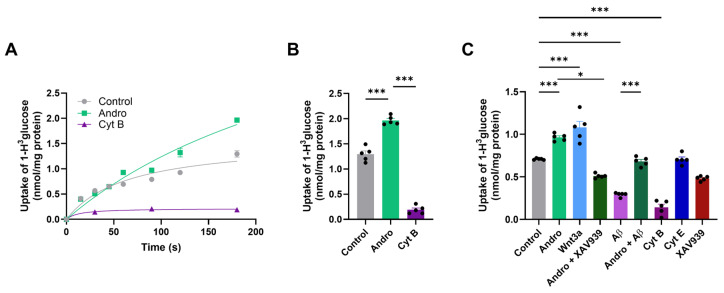
Andro increases the absorption of glucose in cultured astrocytes. (**A**) Time course of [1-^3^H]-2-deoxyglucose uptake in primary astrocyte cultures under control or after treatment with the glucose transport inhibitor cytochalasin B (Cyt B, 20 μM) or Andro (2 μM, 30 min). (**B**) Total glucose uptake measured at 180 s. (**C**) A comparison of the absorption of glucose in astrocytes treated with Andro (2 µM), Wnt3a (100 ng/mL), Aβ_1–42_ oligomers (5 µM), Cyt B and E (20 µM), and XAV939 (2 µM). The mean values ± SEM (*n* = 5, separate cultures) are used to represent the data. Bonferroni’s post hoc test was used after either a one-way ANOVA (**B**,**C**) or a two-way ANOVA (**A**). * *p* < 0.05 and *** *p* < 0.001.

To investigate whether Andro modulates glucose uptake in astrocytes, we measured glucose incorporation in primary astrocyte cultures. First, we study the effect of Andro on cell viability to this we treated the cells with Andro 2 mM, 20 mM and 200 mM by 0–24 h, we observed that in a same manner to previously described previously the 2 μM treatment does not affect the astrocyte viability (Figure S1) [15]. As shown in Figure 1A, Andro treatment (2 μM, 30 min) significantly increased acute glucose uptake, in initial velocity condition (0–180 s) compared with control cells, whereas the glucose transporter inhibitor cytochalasin B (Cyt B, 20 μM) almost completely abolished uptake. The Andro effect on glucose uptake was similar with Andro 20 mM and 200 mM, (Figure S2).

Quantitative analysis of total glucose uptake after 180 s established these findings (Figure 1B). Andro increased glucose incorporation by approximately 25% compared with control cells, whereas Cyt B reduced uptake by more than 80%.

Next, we contrasted Andro with other recognized glucose metabolism modulators. A comparable stimulatory response was achieved by exposure to Wnt3a, which supported Andro’s activation of Wnt/β–catenin signaling. Co-treatment with XAV939 [16] (a well-known intracellular inhibitor of Wnt canonical signaling, Figure 1C) eliminated Andro’s effect on glucose absorption. On the other hand, Aβ oligomer therapy (Aβ_1–42_, 5 mM) significantly reduced glucose absorption, which is in line with the metabolic impairment observed in AD models. By preventing the Aβ-induced decrease, co-treatment with Andro kept glucose uptake at control levels (Figure 1C). As anticipated, Cyt B totally prevented all groups from incorporating glucose, but Cyt E had no effect on glucose absorption.

### 2.2. Andrographolide Selectively Activates the PPP in Astrocytes

**Figure 2:**
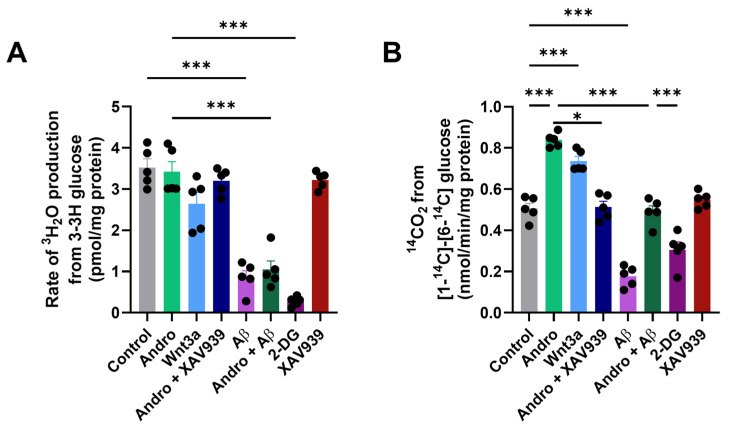
In cultured astrocytes, Andro specifically activates the PPP. (**A**) The rate of [^3^H]_2_O generation from [3-^3^H]-glucose in primary astrocyte cultures under control conditions or following treatment with Andro (2 μM, 30 min), Wnt3a (100 ng/mL), Aβ_1–42_ oligomers (5 μM), XAV939 (2 μM), or Andro + Aβ and 2-DG was used to determine glycolytic flux. (**B**) PPP activity was measured by comparing the synthesis of [^14^C]-CO_2_ from [1-^14^C]-glucose and [6-^14^C]-glucose. Andro significantly raised PPP flow in comparison to control; this effect was like that of Wnt3a and was eliminated by the selective G6PDH inhibitor 2-DG. The mean values ± SEM (*n* = 5 separate cultures) are used to represent the data. Bonferroni’s post hoc test was conducted after one-way ANOVA, * *p* < 0.05 and *** *p* < 0.001.

To determine whether Andro influences downstream glucose utilization, we next examined its effects on glycolytic flux and the PPP in cultured astrocytes. As shown in Figure 2A, Andro treatment did not significantly modify the glycolytic rate compared with control cells. By contrast, exposure to Aβ_1–42_ oligomers markedly reduced glycolytic activity to approximately 30% of control values. Co-treatment with Andro failed to prevent this decline, suggesting that Andro does not primarily enhance glycolysis. As expected, the glycolytic inhibitor 2-DG completely abolished glucose metabolism. The effect of Andro was not affected by the presence of XAV939 (Figure 2A).

We then assessed the oxidative branch of glucose metabolism. Andro significantly increased PPP activity by nearly 60% compared with control conditions (Figure 2B) reaching levels comparable to those elicited by Wnt3a. The stimulation of the PPP was abolished by the presence of 2-DG. Conversely, Aβ_1–42_ oligomers sharply decreased PPP activity, consistent with their reported disruption of astrocytic redox metabolism. Importantly, Andro treatment restored PPP flux in Aβ-treated cultures to near-control levels, revealing a selective activation of this pathway under oxidative stress conditions. By contrast, the stimulation of PPP by Andro was inhibited in the presence of XAV939 to values comparable to control condition (Figure 2B).

We additionally examined the metabolic effects of Andro in C6 glioma cells, a complementary astrocytic model [17]. As shown in Figure S3, Andro increased glucose uptake without enhancing glycolytic flux, while promoting a modest rise in PPP-dependent glucose oxidation and a significant elevation in total GSH content. This metabolic profile closely paralleled the response observed in primary cortical astrocytes, indicating that Andro induces a consistent shift toward PPP-driven redox reinforcement across distinct astroglial systems.

### 2.3. Andrographolide Modulates the Activity of Key Metabolic Enzymes in Astrocytes

**Figure 3:**
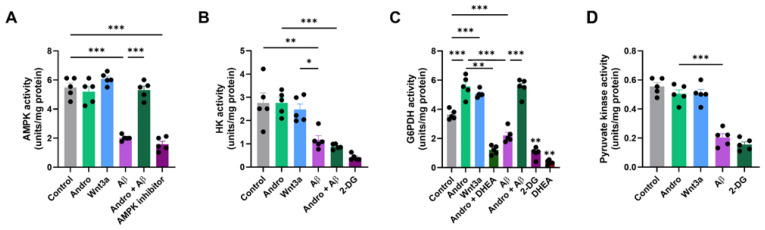
In cultured astrocytes, Andro modifies important regulatory enzymes of glucose metabolism. Following treatment with Andro (2 µM, 30 min), Wnt3a (100 ng/mL), Aβ_1–42_ oligomers (5 µM), Andro + Aβ, or certain enzymatic inhibitors, the activities of important metabolic enzymes were assessed in primary astrocyte cultures. (**A**) When compared to the control, Andro did not significantly alter AMPK activation. (**B**) Andro had no effect on Hk activity, which is the initial stage of glycolysis. (**C**) Following Andro therapy with or without DHEA (25 µM), G6PDH, the PPP’s rate-limiting enzyme, exhibited a substantial increase in activity (*p* < 0.001). (**D**) Andro had no effect on Pk activity, a terminal glycolytic enzyme. The mean values ± SEM (*n* = 5 separate cultures) are used to represent the data. One-way ANOVA followed by Bonferroni’s post hoc test was performed, * *p* < 0.05, ** *p* < 0.01, and *** *p* < 0.001.

We next analyzed the activity of major regulatory enzymes involved in cellular energy metabolism, including AMPK, Hk, G6PDH, and Pk in primary astrocyte cultures. As shown in Figure 3A, Andro treatment did not modify basal AMPK activity compared with control cells. However, Aβ_1–42_ oligomers induced a pronounced inhibition of AMPK activity (approximately 60% reduction). Remarkably, co-treatment with Andro completely prevented this inhibition, restoring AMPK activity to control levels. The positive control Wnt3a, which activates canonical Wnt signaling, elicited a similar protective effect, whereas the selective AMPK inhibitor abolished enzyme activity.

Next, we evaluated the first regulatory step of glucose metabolism through Hk activity (Figure 3B). Andro did not increase Hk activity compared with control. In contrast, Aβ exposure significantly reduced Hk activity to ~40% of control, this was not affected by the Andro co-treatment. As expected, 2-DG, a competitive Hk inhibitor, nearly abolished Hk activity.

In contrast, Andro markedly stimulated G6PDH enzymatic activity, the rate-limiting step of the PPP (Figure 3C). Compared with control cells, both Andro and Wnt3a increased G6PDH activity by nearly 50%, whereas Aβ treatment reduced it by more than 60%. Co-treatment with Andro effectively rescued this decline, restoring activity to basal levels. The stimulatory effect was abolished by 2-DG, confirming the glucose-dependent nature of this response. The co-treatment of Andro with DHEA (a well described inhibitor of G6PDH activity [18]) abolished the stimulatory effect induced by Andro, supporting a central role of the activity of this enzyme (Figure 3C).

Finally, Pk activity, the terminal enzyme of glycolysis, was not significantly modified by Andro or Wnt3a but was strongly reduced after Aβ exposure, as shown in Figure 3D.

### 2.4. Andrographolide Reduces Cellular Energy Charge and Redirects Glucose Metabolism Toward Antioxidant Functions

**Figure 4:**
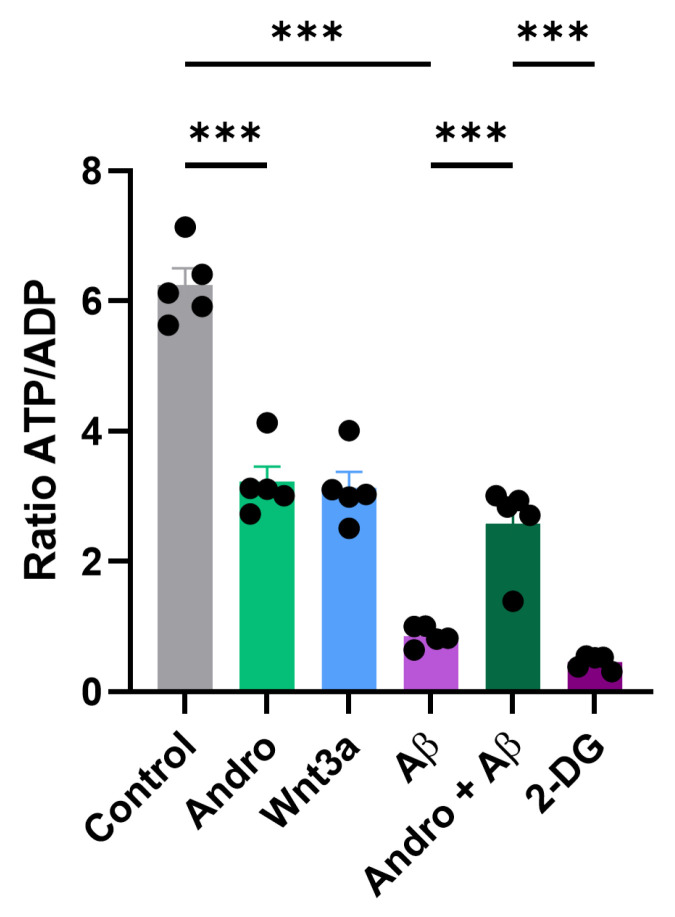
After 30 min of treatment with Andro (2 µM), Wnt3a (100 ng/mL), Aβ_1–42_ oligomers (5 µM), Andro + Aβ, or a metabolic inhibitor, the intracellular ATP/ADP ratio was measured in primary astrocyte cultures. The high ATP/ADP ratio in control astrocytes was indicative of normal energy metabolism. The ATP/ADP ratio was considerably lowered with Andro treatment (*p* < 0.001). The mean values ± SEM (*n* = 5 separate cultures) are used to represent the data. Bonferroni’s post hoc test was conducted after one-way ANOVA, *** *p* < 0.001.

Given the selective activation of the PPP by Andro, we next examined whether this metabolic shift was accompanied by alterations in cellular energy status. To this end, we quantified the ATP/ADP ratio as an indicator of the balance between energy production and consumption in cultured astrocytes. As shown in Figure 4, control astrocytes exhibited a high ATP/ADP ratio, reflecting robust oxidative and glycolytic activity under basal conditions. Treatment with Andro (2 µM, 30 min) significantly decreased this ratio by nearly 50% compared to control. A similar reduction was observed following exposure to Wnt3a. Conversely, Aβ_1–42_ oligomers produced a profound drop in the ATP/ADP ratio, more than 80% reduction, indicative of mitochondrial dysfunction and impaired energy metabolism, as reported in AD models. Co-treatment with Andro partially prevented this decline, maintaining energy charge at intermediate levels relative to Aβ-treated cells. The use of a metabolic inhibitor completely abolished ATP/ADP ratios, confirming assay specificity.

### 2.5. Andrographolide Enhances Antioxidant Capacity and Redox Balance in Astrocytes

**Figure 5:**
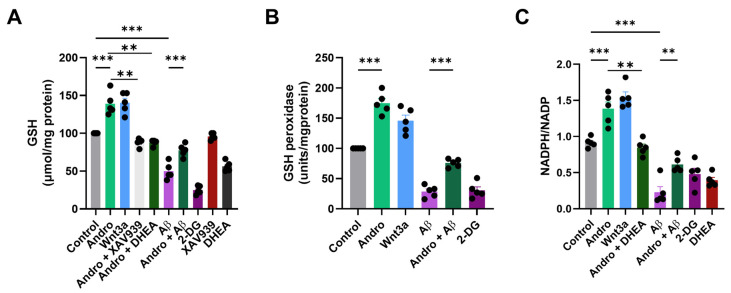
In cultured astrocytes, Andro improves redox equilibrium and antioxidant capability. After primary astrocyte cultures were treated with Andro (2 μM, 30 min), Wnt3a (100 ng/mL), Aβ_1–42_ oligomers (5 μM), DHEA (25 μM), XAV939 (2 μM), Andro + Aβ, or 2-DG, (**A**) total GSH content, (**B**) GPx activity, and (**C**) NADPH/NADP^+^ ratio. When compared to the control, Andro dramatically raised GSH levels and GPx activity (*p* < 0.01, *p* < 0.001). There was a notable increase in the NADPH/NADP^+^ ratio. The mean values ± SEM are used to represent the data (*n* = 5 separate cultures). Bonferroni’s post hoc test was conducted after one-way ANOVA, ** *p* < 0.01, and *** *p* < 0.001.

Next, we analyzed total GSH content, GPx activity, and the NADPH/NADP^+^ ratio, key indicators of cellular redox status. As shown in Figure 5A, Andro treatment significantly increased total GSH levels by approximately 40% compared with control condition, a response comparable to that elicited by Wnt3a. The effect of Andro was partially abolished in presence of Wnt canonical Inhibitor XAV939. In contrast, exposure to Aβ_1–42_ oligomers drastically reduced GSH levels to nearly 50% of control. Importantly, this effect was abolished by inhibition of G6PDH by the co-treatment with DHEA. While we do not claim that the PPP is the only cellular source of NADPH/GSH, our data demonstrate that PPP activity is a contributor to the Andro-dependent increase in GSH under these conditions (Figure 5A).

Similarly, GPx activity was markedly enhanced by Andro and Wnt3a (Figure 5B), showing a 70–80% rise relative to controls. Conversely, Aβ treatment significantly decreased GPx activity, while Andro co-treatment preserved enzymatic activity at near-control levels. The protective effect of Andro was lost in the presence of a PPP inhibitor, reinforcing the link between Andro-induced PPP activation and enhanced antioxidant enzyme function.

To further assess the redox implications of this metabolic remodeling, we measured the NADPH/NADP^+^ ratio, a key determinant of cellular reducing power. As shown in Figure 5C, Andro markedly increased this ratio by ~50% compared to control, mirroring the effect of Wnt3a. Aβ treatment drastically decreased the NADPH/NADP^+^ ratio (*p* < 0.001), but it was partially restored when Andro was co-administered. The increase in NADPH/NADP ratio was abolished by the co-treatment with DHEA (Figure 5C).

### 2.6. Andrographolide Modulates Gene Expression Involved in Glucose Transport, Wnt Signaling, and Inflammation in Astrocytes

**Figure 6:**
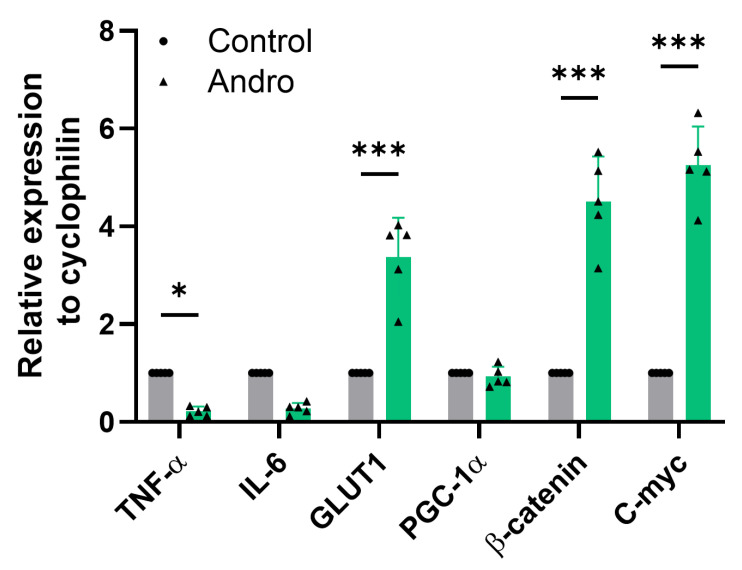
In cultured astrocytes, Andro controls the expression of genes related to metabolism, antioxidants, and inflammation. Quantitative RT-PCR was used to measure the relative mRNA expression levels of TNF-α, IL-6, GLUT1, PGC-1α, β–catenin, and c-Myc in primary astrocyte cultures treated with Andro (2 μM, 30 min) or vehicle (Control). GLUT1, β–catenin, and c-Myc expression were all markedly elevated by Andro. The mean values ± SEM (*n* = 5 separate cultures) are used to represent the data. Bonferroni’s post hoc test was conducted after a two-way ANOVA, * *p* < 0.05, *** *p* < 0.001. **Grey:** Control condition. **Green:** Andro-treated cells.

We next examined the transcriptional response of genes involved in glucose transport, Wnt/β–catenin signaling, mitochondrial regulation, and inflammation in primary astrocytes by quantitative RT-PCR. As illustrated in Figure 6, Andro markedly increased GLUT1 mRNA expression, the predominant glucose transporter in astrocytes, reaching approximately a fourfold elevation relative to control cells. In parallel, the expression of β–catenin and c-Myc, two canonical Wnt target genes, was significantly upregulated, consistent with activation of this signaling pathway.

In contrast, transcript levels of the mitochondrial biogenesis regulator PGC-1α were not affected by Andro treatment. Analysis of inflammatory markers revealed a significant reduction in TNF-α mRNA, whereas IL-6 expression remained unchanged. Accordingly, our conclusions are supported by complementary analyses of enzyme activity, PPP flux, redox state, and antioxidant capacity, which together provide direct evidence of andrographolide-induced metabolic reprogramming in astrocytes.

## 3. Discussion

The current study demonstrated that in cultured mouse astrocytes, Andro, a diterpenoid lactone isolated from *Andrographis paniculata*, induces a metabolic shift that prioritizes antioxidant defense above ATP synthesis. We showed that Andro increases glucose uptake, preferentially stimulates the PPP by activating G6PDH, and increases NADPH production, GSH content, and GPx activity by a combination of biochemical, enzyme activity, pharmacological, and molecular investigations. At the same time, Andro lowers ATP/ADP ratios, suggesting that the PPP receives the increased glucose flux instead of glycolysis. These findings collectively imply that Andro reprograms astrocyte metabolism toward redox homeostasis and anti-inflammatory defense, offering a molecular foundation for its previously reported neuroprotective effects in AD and kindred neurodegenerative disease models.

Astrocytes are essential for preserving the energy balance of the brain [4,5]. Astrocytes have a more adaptable metabolic profile than neurons, which primarily rely on glycolysis and oxidative phosphorylation to meet their high ATP demands [19,20,21]. Because of their adaptability, they can direct glucose into either the PPP or glycolysis, depending on what the cell requires at any time [22,23]. Because it produces NADPH, a crucial reducing agent that supports antioxidant systems like the GSH–GPx axis, the PPP is particularly crucial for controlling oxidative stress [6,24].

Our results show that Andro strengthens this adaptive capacity by selectively activating the PPP while leaving glycolytic enzymes largely unchanged. This metabolic shift enables astrocytes to support their antioxidant defenses, even if it comes at the cost of reduced ATP production, as reflected by the lower ATP/ADP ratio following Andro treatment.

This pattern resembles a metabolic preconditioning response, in which mild energy reduction is compensated by enhanced redox capacity, preparing cells to cope with oxidative insults. A similar shift has been described in neurons under Wnt3a stimulation, which increases glucose uptake but directs its utilization toward biosynthetic and antioxidant pathways rather than oxidative ATP production [15]. The present findings extend this concept to glial cells, revealing that Andro activates a comparable regulatory program in astrocytes, likely mediated by the Wnt/β–catenin signaling cascade. This metabolic shift may be particularly relevant in the context of AD, where inflammatory and oxidative insults predominantly target neuronal populations.

Previous studies examining the metabolic actions of Andro have focused mainly on neurons, where these pathways generally enhance glycolysis and ATP production. In contrast, reports addressing glial metabolism remain scarce and have not described a selective redirection of glucose toward the PPP [5,25,26]. Our findings reveal a fundamentally different metabolic phenotype in astrocytes: despite increasing glucose uptake, Andro does not augment glycolysis but instead activates G6PDH and boosts PPP-derived NADPH generation, particularly under Aβ exposure. Interestingly, we observed that the presence of a downstream Wnt canonical signaling inhibitor could not abolish the effect of Andro effects. However, the effect of Andro on astrocytes metabolism was in part dependent of the activation of G6PDH since the co-treatment with the G6PDH inhibitor was able to abolish the Andro stimulation.

Previous studies have established that Andro acts as a non-ATP competitive inhibitor of GSK-3β, leading to stabilization and nuclear accumulation of β–catenin and activation of Wnt target genes [27,28]. The current results reinforce the functional relevance of this mechanism in astrocytic metabolism. Our exploratory studies, using qrt-PCR, showed that Andro stimulate an upregulation of β–catenin and c-Myc, both canonical Wnt signaling targets, suggesting that this pathway could be associated with the Andro effects in astrocytes, like previously described in neurons [27,29]. Previously has been described that the Wnt/β–catenin pathway influences how cells balance ATP production with antioxidant metabolism, in part by regulating G6PDH expression and activity [30]. In this context, the rise in PPP activity observed after Andro treatment may reflect a downstream consequence of Wnt pathway stimulation.

The PPP-derived NADPH is essential for the maintenance of the reduced form of GSH, which acts as one of the principal intracellular antioxidants in brain cells [30,31]. Our findings demonstrate that Andro treatment elevates both total GSH content and increases GPx activity, these results could associate the PPP activation to the support of antioxidant defenses. This is consistent with previous reports showing that pharmacological activation of G6PDH or increased NADPH availability protects astrocytes and neurons from oxidative damage [24,32]. By enhancing NADPH production, Andro could promote the regeneration of GSH from GSSG via glutathione reductase, thus sustaining ROS detoxification through GPx activity. Interestingly, the increase in NADPH/NADP^+^ ratio induced by Andro occurred alongside a marked reduction in ATP/ADP ratio, indicating a metabolic trade-off between bioenergetic and antioxidant outputs. This reciprocal regulation could be a hallmark of adaptive redox metabolism in astrocytes, which often sacrifice ATP yield to maintain ROS homeostasis, particularly under pathological stress. In this context, the Andro-induced activation of the PPP may represent a protective metabolic reprogramming aimed at preserving cellular redox integrity rather than energetic efficiency. Although these results should be interpreted with caution. Lower energy charge can also reflect acute metabolic stress, and our in vitro model does not allow us to fully distinguish between adaptive and stress-related components. Thus, we consider the ‘preconditioning-like’ interpretation as a tentative explanation rather than a definitive mechanism, and future studies will be required to clarify this distinction [33,34].

The close relationship between cellular metabolism and inflammation offers a useful lens through which to interpret our findings. When the PPP is activated and NADPH levels rise, cells generally become better equipped to handle oxidative stress, which in turn can dampen pro-inflammatory signaling. In our model, the Andro-induced increase in the NADPH/NADP^+^ ratio may help limit ROS-dependent activation inflammation effectors like NF-κB or TNF-α and the increased y in the GSH levels could be part of this signal to promote an antioxidative defense [9,35].

Previous work has demonstrated that Andro could attenuate clinical brain manifestations of AD and act as a preventive agent against brain deterioration. AD is characterized by early metabolic dysfunction, oxidative stress, and chronic neuroinflammation [36,37]. Astrocytic glucose metabolism plays a central role in sustaining neuronal energy and redox balance, and its impairment has been implicated in disease pathogenesis [10]. The current data identify Andro as a potential modulator of these key processes.

By stimulating glucose transport and the subsequent activation of the PPP would increase NADPH availability, reinforce antioxidant defenses and counteract the elevated oxidative stress typical of AD brains. These mechanisms collectively align with prior in vivo findings showing that Andro administration improves synaptic function, reduces amyloid deposition, and prevents cognitive deficits in transgenic AD mouse models [28,38].

Although our results point to a metabolic program that could be beneficial in the context of AD, it is important to emphasize that these findings derive from an acute in vitro astrocyte model. Thus, the mechanistic link to AD should be viewed as inferential rather than directly demonstrated. Our interpretation is based on the convergence between the metabolic shift observed here and previous in vivo studies reporting reduced oxidative stress, enhanced Wnt signaling, and improved cognitive outcomes after Andrographolide treatment. The present data therefore do not establish a causal role in AD pathology but instead offer a glial mechanism that may contribute to the neuroprotective actions described in animal models. In this context, the Andro-induced decrease in ATP/ADP ratio should not be viewed as detrimental but rather as indicative of a protective metabolic shift. In neurodegenerative diseases, where energy metabolism is chronically compromised, the capacity to redirect glucose toward antioxidant defense may prevent irreversible oxidative damage. Therefore, promoting PPP activation in astrocytes could represent a novel strategy to enhance brain resilience against neurodegeneration.

In summary, our findings point to a role for Andro that had not been clearly appreciated before: it acts as a metabolic regulator in astrocytes, shaping how these cells maintain their redox balance. Andro increases glucose uptake and channels it preferentially into the PPP, which in turn strengthens NADPH-dependent antioxidant defenses and lowers inflammatory activity. This pattern differs from its effects in neurons, where Andro tends to boost glycolysis and energy recovery, highlighting the distinct metabolic responses of each cell type. These insights also help refine our understanding of why Andro shows neuroprotective properties in AD models. Its benefits likely extend beyond direct support to neurons and involve a broader reorganization of astrocytic metabolism that creates a microenvironment more capable of buffering oxidative and inflammatory stress. Given that impairments in astrocyte function appear early in AD pathology, strategies that target glial metabolism may complement existing therapeutic approaches and open new avenues for intervention.

These findings suggest that adjusting astrocytic metabolism pharmacologically may work alongside neuron-focused treatments in AD, offering a potential complementary, glia-centered strategy to strengthen brain resilience. Moving forward, it will be important to clarify the molecular steps that connect Andro-driven Wnt activation with the regulation of G6PDH, as well as to investigate how prolonged treatment influences metabolic communication between astrocytes and neurons in living systems.

It will also be valuable to examine whether Andro acts synergistically with other metabolic or antioxidant agents, as such combinations could help restore disrupted energy metabolism and redox balance more effectively. Overall, our data highlight Andro as a distinctive natural molecule that brings together metabolic reprogramming with antioxidant and anti-inflammatory actions in astrocytes. This integrated profile strengthens its promise as a therapeutic candidate for neurodegenerative diseases, especially those marked by profound metabolic and redox dysfunction, such as AD.

## 4. Materials and Methods

### 4.1. Ethics Statement

The Committee for Ethics of Animal Experiments approved the experiments, which were carried out in accordance with the Guidelines for Animal Experiments, P. Universidad Católica de Chile (CBB-166/2011), and the Manual of Biosafety Standards and Associated Risks, CONICYT 2009.

### 4.2. Primary Cultures of Cortical Astrocytes

A 1-day postnatal C57BL/6 mouse was used to create astrocyte cultures. The cerebral cortex was treated with 0.25% *w*/*v* trypsin for 15 min at 37 °C. They were then mechanically broken down into MEM supplemented with 10% *v*/*v* fetal bovine serum (FBS), 2 mM glutamine, 100 U/mL penicillin, 100 μg/mL streptomycin, 2 mM glucose, and 2.5 μg/mL fungizone. The cells were planted at a density of 2 × 10^4^ cells/cm^2^ in culture plates. Astrocytes were employed in all the studies after being kept in culture for 30 days.

### 4.3. Cells Treatment

Before treatment, the astrocytes were maintained in culture medium without FBS for 30 min and then incubated for several times with: Andro (2 mM, for several times), recombinant Wnt3a (100 ng/mL, for 3 h), Cytochalasin B (Cyt B, an inhibitor of GLUTs, 20 μM, for 30 min) and Cytochalasin E (Cyt E, controls the action of Cyt B, 20 μM, for 30 min, a concentration and exposure time that do not affect cytoskeletal organization [39,40,41], 2-deoxyglucse (2-DG, a competitive inhibitor of Hk, 7 mM, for 30 min), Compound C (CC, inhibitor of AMPK, 10 mM), XAV939 (downstream inhibitor of canonical Wnt signaling, 2 μM), dehydroepiandrosterone (DHEA, an uncompetitively inhibitor of G6PDH, 25 μM) and Ab_1–42_ (peptide corresponding to wild-type human Ab, 5 μM, 12 h). After treatment, the cultures were processed according to the respective protocol.

### 4.4. Glucose Uptake Analysis

Following two rounds of washing the corresponding treatment cultures with incubation buffer (135 mM NaCl, 15 mM HEPES, 5 mM KCL, 1.8 mM CaCl_2_, and 0.8 mM MgCl_2_), 1–1.2 μCi of Deoxy-D-glucose, 2-[1,2-^3^H(N)] ([^3^H]-2-DG) was added multiple times. After stopping the uptake of glucose with detention buffer (add 0.2 mM HgCl_2_ to incubation buffer, pH 7.4), cells were lysed in 1 mL of lysis buffer (10 mM Tris-HCL and 0.2% SDS, pH 8.0). Three milliliters of scintillating solution were then added to the cell lysates, and radioactivity was measured using a liquid scintillation counter (TriCarb 2900TR analyzer).

### 4.5. Determination of the Glycolytic Rate

As previously mentioned, glycolytic rates were calculated [42]. In short, primary cultures were put in tubes with 5 mM glucose and then twice washed in Krebs–Henseleit solution (11 mM Na_2_HPO_4_, 122 mM NaCl, 3.1 mM KCl, 0.4 mM KH_2_PO_4_, 1.2 mM MgSO_4_, and 1.3 mM CaCl_2_, pH 7.4) with the proper amount of glucose. After equilibration in 0.5 mL of Hank’s balanced salt solution/glucose (cat: 14025076, ThermoFisher, Waltham, MA, USA) at 37 °C for 30 min, 0.5 mL of Hank’s balanced salt solution containing various concentrations of [3-^3^H] glucose (cat: NET331C250UC, PerkinElmer, Waltham, MA, USA) was added, with a final specific activity of 1–3 disintegrations/min/pmol (~1 mCi/mmol). After that, 100 μL aliquots were moved to a different tube, put inside a sealed scintillation vial with 0.5 mL of water, and incubated for 48 h at 45 °C. The tube was taken out of the vial, the scintillation mixture was introduced, and the ^3^H_2_O content was measured by counting over a 5 min period following this va-por-phase equilibration stage.

### 4.6. Measurement of AMPK, Pk, G6PDH and Hk Activity

Primary astrocyte cultures were exposed for 30 min to Andro or the indicated activators/inhibitors and subsequently washed with PBS. Total and phosphorylated AMPK (Thr172) levels were quantified using an ELISA-based approach following the manufacturer’s instructions, with fluorescence detection performed at the appropriate excitation and emission wavelengths for each AMPK form. Pk activity was assessed using a commercial colorimetric assay. Hk activity was determined from cell lysates obtained after trypsinization, controlled lysis, and differential centrifugation. The soluble fraction was incubated with a glucose-containing reaction buffer, and enzymatic activity was estimated by spectrophotometric detection of NADPH production. For G6PDH activity measurements, treated cells were lysed under cold conditions and subjected to sequential centrifugation to isolate the cytosolic fraction. This fraction was incubated with glucose-6-phosphate and ATP, and NADPH generation was monitored as a functional readout of pentose phosphate pathway activity.

### 4.7. Quantification of ADP, ATP Content and NADPH/NADP^+^

An ATP determination kit (cat: A22066, Invitrogen/Molecular Probes, Waltham, MA, USA) was used to determine the amount of ATP in primary cultures of astrocytes that had been treated with activators or inhibitors. An ADP Assay Kit (cat: ab83359, Abcam, Cambridge, UK) was used to measure the ADP levels in slices in accordance with the manufacturer’s instructions. A commercial colorimetric assay (#MAK038, Sigma-Aldrich, St. Louis, MO, USA) was used to determine the NADPH/NADP+ ratio in accordance with the manufacturer’s instructions, as previously mentioned.

### 4.8. Measurement of Glucose Oxidation Through the Pentose Phosphate Pathway (PPP)

Glucose oxidation through the pentose phosphate pathway (PPP) was quantified following established radiometric methods, based on the differential decarboxylation of [1-^14^C]-glucose and [6-^14^C]-glucose. Briefly, [1-^14^C]-glucose releases ^14^CO_2_ both in the 6-phosphogluconate dehydrogenase reaction of the PPP and in the Krebs cycle, whereas [6-^14^C]-glucose is decarboxylated exclusively in the Krebs cycle. Thus, PPP-derived glucose oxidation was calculated as the difference in ^14^CO_2_ production between both labeled substrates. Cells were washed with ice-cold PBS, harvested, and immediately transferred into O_2_-saturated Krebs–Henseleit buffer (122 mM NaCl, 3.1 mM KCl, 0.4 mM KH_2_PO_4_, 11 mM Na_2_HPO_4_, 1.2 mM MgSO_4_, 1.3 mM CaCl_2_, pH 7.4). The cellular suspension was placed in Erlenmeyer flasks containing an additional 0.5 mL of Krebs–Henseleit buffer supplemented with either 0.5 μCi D-[1-^14^C]-glucose or 2 μCi D-[6-^14^C]-glucose, together with unlabeled D-glucose to reach a final concentration of 5.5 mM.

Each flask was fitted with a central well containing an Eppendorf tube with 500 μL benzethonium hydroxide to trap released ^14^CO_2_. Flasks were flushed with O_2_ for 20 s, sealed with rubber caps, and incubated in a shaking water bath at 37 °C for 60 min. Reactions were terminated by injecting 0.2 mL of 1.75 M HClO_4_ into the main chamber to release dissolved CO_2_. Shaking was continued for an additional 20 min to ensure complete trapping of ^14^CO_2_. The radioactivity accumulated in the benzethonium hydroxide was quantified by liquid scintillation counting. PPP-dependent glucose oxidation was obtained by subtracting the ^14^CO_2_ values generated from [6-^14^C]-glucose from those generated from [1-^14^C]-glucose. Radiolabeled substrates were purchased from PerkinElmer (MA, USA) [31,42].

### 4.9. RNA Extraction, RT-PCR and qPCR

TRIzol was used to extract total RNA from cultures in accordance with the manufacturer’s instructions. A spectrophotometer was used to measure the quantities of RNA samples at 260 nm absorbance. In a denaturing agarose gel, the integrity of the RNA was confirmed. As directed by the manufacturer, Superscript IV random primers were used to synthesize cDNA from 500 ng of total RNA. Using the SYBRTM green PCR master Mix (Thermofisher, Waltham, MA, USA) and the following software, the cDNA was examined by qPCR: 20 s at 95 °C, 40 cycles of 5 s at 95 °C and 30 s at 60 °C, followed by a termination step between 55 °C and 95 °C (to confirm the amplification’s specificity). The following primers were utilized for qPCR: GLUT1 (foward: 5′-ATGGATCCCAGCAGCAAGAAG-3′, reverse: 5′-AGAGACCAAAGCGTGGTGAG-3′; c-Myc (foward: 5′-GGAGTGGTTCAGGATTGGGG-3′, reverse: 5′-GGGTAGCTTACCAGAGTCGC-3′), IL-6 (foward: 5′-CAACGATGATGCACTTGCAGA-3′, reverse: 5′-GTGACTCCAGCTTATCTCTTGGT-3′, b–catenin (foward: 5′-TCCTTCACGCAAGAGCAAGTA-3′, reverse: 5′-CGCACCATGCAGAATACAAATGA-3′), TNF-a (foward: 5′-TGATCGGTCCCCAAAGGGAT-3′, reverse: 5′-TGTCTTTGAGATCCATGCCGT-3′), PGC-1a (foward: 5′-AGCCGTGACCACTGACAACGAG-3′, reverse: 5′-GCTGCATGGTTCTGAGTGCTAAG-3′), housekeeping cyclophilin (forward: 5′-TGGAGATGAATCTGTAGGAGGAG-3′, reverse: 5′-TACCACATCCATGCCCTCTAGAA-3). Data were analyzed using the comparative ΔCT method, as previously described [43,44].

### 4.10. Glutathione Determination in Astrocytes

The cells were pre-incubated with these molecules at a concentration of 1 mM to assess the levels of GSH. After that, the cells were cleaned with PBS and separated from the growth plates using 1% *v*/*v* sulfosalisylic acid. The cell lysate was centrifuged for five minutes at 4 °C at 13,000× *g*. Total GSH levels (GSH + 2×GSSG) were measured using the supernatant. Then, 96-well plates containing 0.1 M phosphate, 0.2 M EDTA, 0.3 mM 6,6′-Dithionitro-3,3′-benzoic acid, 0.4 mM β-Nicotinamide adenine dinucleotide 2′-phosphate, and 1 U/mL GSH reductase were used for quantification. Lastly, the absorbance was measured every 15 s for 2.5 min at 405 nm. To quantify GSSG, equal volumes of the cell supernatant and 2-vinylpyridine were mixed, the mixture was incubated for 1 h, then 1 mL of reaction buffer was added, and the absorbance was measured at 405 nm for 5 min (every 30 s).

### 4.11. Statistical Analysis

All experiments were performed using five fully independent biological replicates, each derived from distinct primary astrocyte cultures. Within each biological replicate, measurements were conducted in technical triplicates, and the mean value of each replicate was used for statistical comparisons. Data are presented as mean ± standard error of the mean (SEM). Depending on the experimental design, statistical comparisons were performed using one-way or two-way analysis of variance (ANOVA) followed by Bonferroni’s post hoc test to correct for multiple comparisons. Before running ANOVAs, we verified normality (Shapiro–Wilk test) and homogeneity of variance (Levene’s test) using Prism (GraphPad, USA, version 10.6.1). In cases where assumptions were not met, data were log-transformed, and the analysis repeated to ensure robustness. The significance thresholds were set at *p* ≤ 0.05 (*), *p* ≤ 0.01 (**), and *p* ≤ 0.001 (***). Potential outliers were evaluated using the ROUT method (Q = 1%) implemented in Prism; no outliers were detected or removed from the analyses. All statistical settings (alpha = 0.05, confidence interval = 95%) followed Prism’s standard parameters. The complete statistical framework has been clarified to enhance reproducibility and transparency.

## 5. Conclusions

The conclusions of this study can be emphatically summarized as follows:

1. Andro tended to increase glucose uptake in cortical astrocytes.
2. The compound selectively enhances the PPP by enhancing G6PDH activity, increasing NADPH production and redox capacity.
3. Andro increase total GSH levels and GPx activity, improving antioxidant defense and maintaining redox homeostasis.
4. A reduction in the ATP/ADP ratio indicated a metabolic shift favoring antioxidant protection over energy production.
5. Overall, these findings identify astrocytic metabolic modulation as a novel mechanism underlying Andro’s neuroprotective actions in neurodegenerative disease models.

## Data Availability

Data will be made available upon reasonable request due to institutional restrictions.

## Supplementary Materials

The following supporting information can be downloaded at: https://www.mdpi.com/article/10.3390/ph19010133/s1, Figure S1: Study of Andro treatment on cell viability; Figure S2: Study of uptake of glucose after several times of treatment with Andro; Figure S3: Andro enhances glucose uptake and antioxidant capacity in C6 glioma cells used as an astrocytic model.

## Abbreviations

The following abbreviations are used in this manuscript:

AD	Alzheimer disease
Andro	Andrographolide
AMPK	AMP-activated protein kinase
Aβ	Beta-amyloid peptide
BBB	Blood–brain barrier
Cyt B	Cytochalasin B
Cyt E	Cytochalasin e
2-DG	2-deoxyglucose
GLUT1	Glucose transporter 1
G6PDH	Glucose-6-phosphate dehydrogenase
GSH	Glutathione
GPx	Glutathione peroxidase
GSK-3β	Glycogen synthase kinase-3 beta
Hk	Hexokinase
NADPH	Adenine dinucleotide phosphate
PPP	Pentose phosphate pathway
ROS	Reactive oxygen species
