# Supplementary material for: Metabolic Reprogramming by Andrographolide: Enhanced Pentose Phosphate Pathway and Antioxidant Capacity in Cortical Astrocytes
Source: Pharmaceuticals (Basel). 2026 Jan 12;19(1):133. doi: 10.3390/ph19010133 (PMC12845274; doi:10.3390/ph19010133)
Supplement: Supplementary file 1 [file pharmaceuticals-19-00133-s001.zip › pharmaceuticals-4014808-supplementary.pdf]

Supplementary 1

Cell viability. We treated the astrocytes for several times (0-24h) with several doses of Andro 2 μM (A), 20 μM (B) and 200 μM (C).

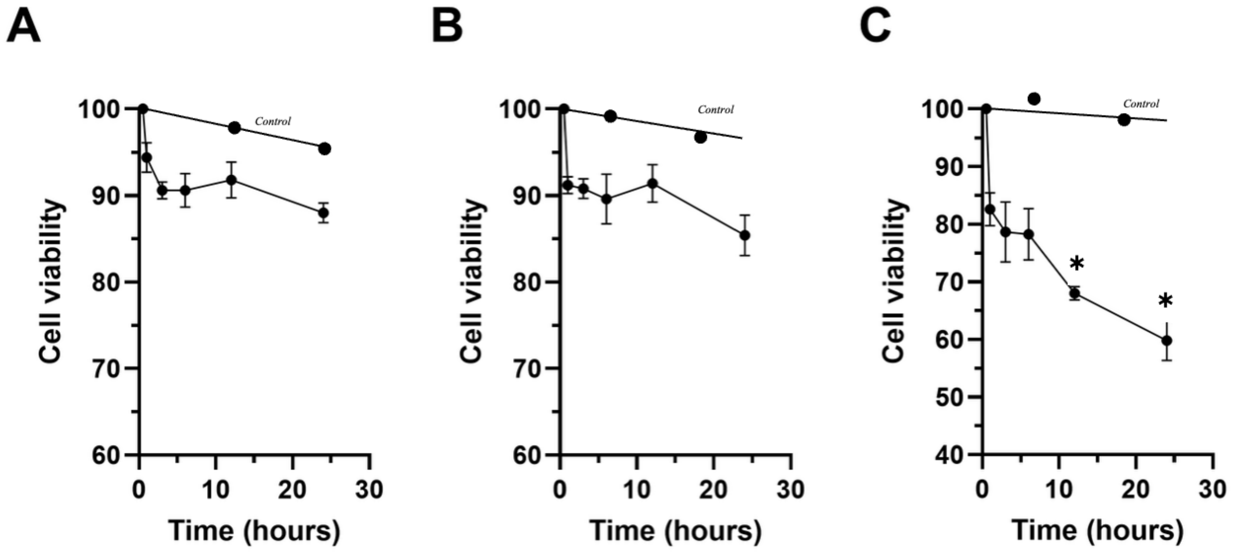

Figure legend. *Study of Andro treatment on cell viability*

We treated the cell with Andro for several times (0-24h) in presence of Andro 2 μM (A), 20 μM (B) and 200 μM (C). With 2 μM of Andro the cell viability after 24 h of treatments was near to 89%. After 1h of treatment the viability was near of 96%. These data support that our treatment of 30 min do not decrease the cell viability of astrocytes,

Data are expressed as mean ± SEM (n = 5 independent cultures). Statistical comparisons were performed using unpaired two-tailed Student's t-test.

Supplementary 2

Glucose uptake after 60 or 120 of treatment with Andro 2 μM.

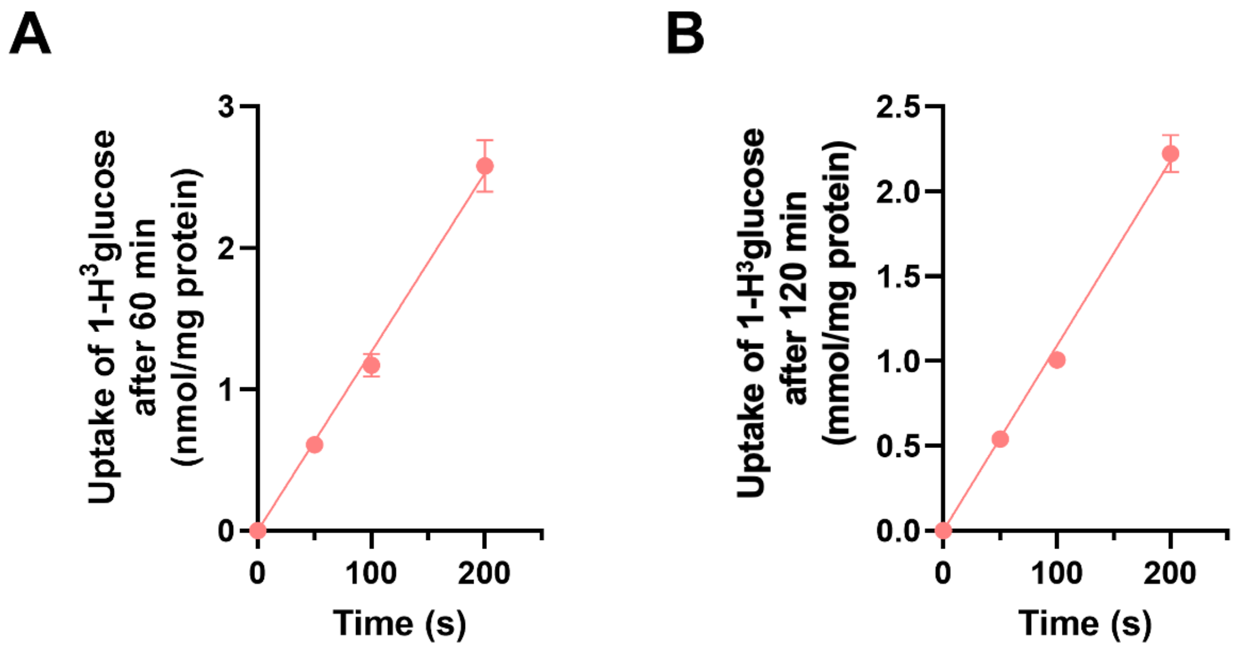

Figure legend. *Study of uptake of glucose after several times of treatment with Andro*

We treated the cell with Andro 2 μM for 60 (A) or 120 min (B). After Andro treatment we study in initial velocity the acute uptake of glucose. We observed that in both conditions the presence of Andro stimulate the glucose uptake.

Data are expressed as mean ± SEM (n = 5 independent cultures). Statistical comparisons were performed using unpaired two-tailed Student's t-test.

Supplementary 3

Protocol of C6 cells

The C6 cells were seeded in 55 cm^2 Petri dishes and maintained in 10 mL of DMEM culture medium without phenol red and glucose with 10% Fetal Bovine Serum (FBS), 4 mM glutamine and 1% penicillin, under standard conditions of 5% CO_2 and a temperature of 37 °C for 24 h to achieve cell fixation. After we reach to cell confluence, we treated the cells with Andro (2 μM) by 30 min. All the experiments performed in these cells were following the protocols described in the manuscript.

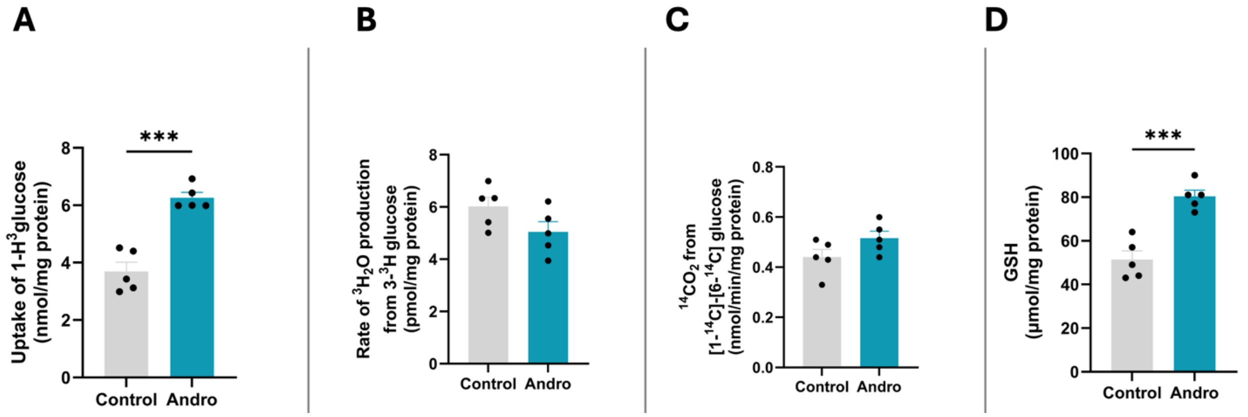

Figure legend. *Andro enhances glucose uptake and antioxidant capacity in C6 glioma cells used as an astrocytic model.*

(A) Uptake of [1-^{3}H]-glucose was quantified in C6 cells under control conditions or after treatment with Andro (2 μM, 30 min). Andro significantly increased glucose incorporation compared with control.
(B) Glycolytic rate determined by the production of ^3H_2O from [3-^{3}H]-glucose showed no significant differences between groups.
(C) Glucose oxidation through the PPP, assessed by the differential release of $^{14}CO_2$ from [1-^{14}C]-glucose vs. [6-^{14}C]-glucose, tended to increase in Andro-treated cells.
(D) Total GSH content was significantly elevated following Andro exposure, indicating enhanced antioxidant capacity.
Data are expressed as mean ± SEM (n = 5 independent cultures). Statistical comparisons were performed using unpaired two-tailed Student's t-test. ***$p < 0.001$ versus control.
